# Dental color measurement to predict DNA concentration in incinerated teeth for human identification

**DOI:** 10.1371/journal.pone.0196305

**Published:** 2018-04-26

**Authors:** Leticia Rubio, Jose Manuel Sioli, Maria Jesús Gaitán, Stella Martin-de-las-Heras

**Affiliations:** 1 Department of Forensic Medicine, University of Malaga, Malaga, Spain; 2 Department of Forensic Medicine and Forensic Dentistry, University of Granada, Granada, Spain; College of Agricultural Sciences, UNITED STATES

## Abstract

Teeth exposed to thermal stress can shed light on the identification of incinerated individuals and on the circumstances of the fire. Changes in the color of burned teeth can provide information on structural changes and the temperature of exposure. The objective of this study was to correlate color modifications with the concentration of human DNA in teeth burned at different temperatures. Spectrophotometry was used to measure the color of 40 teeth heated at temperatures of 100, 200, and 400°C for 60 min. DNA was extracted by phenol-chloroform extraction and quantified by real-time quantitative PCR using the Quantifier human DNA quantification kit. Preliminary results indicated an association of higher temperature with changes in colorimetric variables and a decrease in DNA concentrations. A significant positive correlation was found between luminosity values and DNA concentration (*r* = 0.4727, *p* = 0.0128) and between chromaticity a* values and DNA concentration (*r* = 0.4154, *p* = 0.0250). Spectrophotometry analysis of the color of burned teeth may predict the feasibility of extracting human DNA for identification purposes.

## Introduction

The identification of incinerated human remains poses a major challenge to forensic laboratories and is more difficult with higher intensity and longer duration of the fire. Teeth are the hardest structures in the human body, and their pulp is well protected by dentin, enamel, and cementum, explaining their frequent use for obtaining DNA from extremely damaged or degraded human remains (e.g., due to long post-mortem delay, major catastrophe, or high-temperature fire, among others) [1,2].

Exposure to high temperatures evaporates water and organic material, leading to the contraction of dental tissues and changing the structure, shape, and color of teeth. Techniques employed to study these alterations include radiography [3], histology [4,5], scanning electron microscopy [6], and spectrophotometry [7]. The survival of dental DNA after heating is highly dependent on the temperature and duration of the exposure and the type of tooth [8,9]. In general, it has been found that dental DNA can withstand temperatures up to 400°Celsius (C) for one hour, although the quantity and quality of the genetic material obtained are superior at lower temperatures and shorter exposure times, making forensic identification easier. Most researchers have reported that it is very difficult to extract DNA from teeth after their exposure to 500°C [10–12]. In certain circumstances (e.g., intentional incinerations, or accidents in remote locations, etc.), human remains can be exposed to high temperatures for prolonged time periods.

Heat-induced macroscopic and DNA changes in teeth have been described but not the relationship between these parameters. Therefore, the objective of this study was to analyze the correlation between color modifications and DNA concentrations in teeth burned for one hour at different temperatures, exploring whether the presence of genetic material for identification purposes can be predicted by the external appearance of the tooth.

## Materials and methods

### Ethics statement

Written informed consent was obtained from each participant after a complete description of the study. All the participants had the opportunity to discuss any questions or issues. The study and protocols for recruitment were approved by the Human Research Ethics Committee of the University of Malaga (Approval number: CEUMA 2013-0048-H) in accordance with the “Ethical Principles for Medical Research Involving Human Subjects” adopted in the Declaration of Helsinki by the World Medical Association (64th WMA General Assembly, Fortaleza, Brazil, October 2013), Recommendation No. R (97) 5 of the Committee of Ministers to Member States on the Protection of Medical Data (1997), and Spanish data protection act (*Ley Orgánica 15/1999 de Protección de Datos*, LOPD).

### Teeth sampling and preparation

Forty healthy erupted permanent teeth, twenty from male and twenty from female patients, were collected from dental clinics in Malaga (Spain) after their extraction for valid clinical reasons. Five teeth from male patients and five from female patients were randomly assigned to one of four groups, recording the age of the patient in each case, for a 60-min exposure to temperatures of 100, 200, or 400°C or for non-exposure (control group). The mean±standard deviation (SD) age of the patients was 43.1±4.54 yrs for the group exposed at 100°C, 43.7±4.79 yrs for that exposed at 200°C, 44.2±3.57 yrs for that exposed at 400°C, and 38.5± 5.25 yrs for the control group.

Teeth were washed with distilled water post-extraction, cleaning their external surface with curettes to remove any other material, and were then dried in controlled conditions of 21°C and 65% humidity before dispatch to the laboratory.

In the laboratory, teeth were washed for 1.5 minutes in 10% sodium hypochlorite solution, repeatedly rinsed with water to remove any remaining bleach, and then irradiated for 10 min with 256-nm Ultraviolet light (Telstar Mini-V/PCR, Telstar Industrial S.L., Terrassa, Spain).

### Incineration

Teeth were placed in individual crucibles and heated for 60 min in a pre-heated muffle furnace (Nabertherm LT 40/12, Nabertherm GmbH, Germany) at temperatures of 100, 200, or 400°C. The ten control teeth were not heated and were stored at room temperature (21°C).

### Morphological analysis

Visual evaluation of structural and color alterations was performed by an expert observer using natural light under 2.5X magnification (Orascoptic®, Middleton, Wisconsin, USA). Visual inspection was complemented by photographs taken with a Canon 500D® DSLR camera (Canon Inc. Tokyo, Japan) equipped with 100 mm macro lens and Canon MR-14 Ex®annular flash (Canon Inc,). Images were viewed using Adobe Camera Raw version 7.3® (Adobe Systems Inc., San Jose, CA) in zoom mode up to 100%.

### Color measurement

The color of the vestibular tooth surface was evaluated by spectrophotometry [7] using a portable contact Spectro-color® device (Dr Lange, Keison Products Co, England) with 8-mm measuring tip in analysis mode. Colorimetric analysis of fragmented samples was performed on the outer surface of the enamel. Device parameters were D65 for illuminant conditions and 8° for standard observer. The spectrophotometer was calibrated before each measurement session, and the mean of three measurements was considered for each tooth.

Measurements were taken of *Comission Internationale d'Eclairage* (CIELAB) color parameters (L*, a*, b*) and the luminance (Y; units: cd/m^2^; candles/square meter). L* value is a measure of the lightness of an object on a scale from 0 (black) to 100 (white). Chromaticity a* is a measure of redness (positive a*) or greenness (negative a*), and chromaticity b* is a measure of yellowness (positive b*) or blueness (negative b*). The a* and b* coordinates approach zero for neutral colors (white, grays) and increase in magnitude for more saturated or intense colors. The whiteness and yellowness of teeth were evaluated by calculating whiteness (WI) and yellowness (YI) indexes as proposed by the American Society of Testing Materials (ASTM) [13] and according to the tristimulus colorimetry system (X,Y,Z) established by the CIE (International Commission on Illumination) in 1931 [14], using the formulas:

WI=4Z%−3Yrel

YI=100(1−0.847Z/Yrel)

where *Z*% is calculated as (*Z/Z*_*n*_) 100, *Z* is the tristimulus *Z* of the sample, and *Z*_*n*_ the tristimulus *Z* of the white standard, while *Y*_*rel*_ is the luminance factor calculated as *Y*_*rel*_ = 100 (*Y/Y*_*n*_), where *Y* is the luminance of the sample and *Y*_*n*_ is the luminance of the white standard.

### DNA extraction and quantification

Samples were pulverized in liquid nitrogen with a 6770 Freezer Mill (SPEX CertiPrepFreezerMill, Stanmore, London, UK). Next, 500 mg of dental powder was transferred to 1.5-mL conical tubes, demineralized with 0.5 M EDTA (pH 8.0) plus 35 μL 10% (wt/vol) sodium dodecylsulfate and 100 μL proteinase K (20 mg/mL) in order to lyse cell and nucleus walls and denature proteins. Samples were incubated at 37°C for 12–18 hours. Then, 50 μL proteinase K (20 mg/mL) was added and the samples were again incubated at 37°C for 2 h followed by their centrifugation. Each sample was extracted three times with phenol/chloroform/isoamyl alcohol (25:24:1). The upper aqueous layer was transferred to a 1.5-mL tube and extracted once with chloroform/isoamyl alcohol (24:1), washed with distilled water three times in a Centricon-100 concentrator (Centricon-100, Millipore, Bedford, MA), and concentrated with 1X Tris-EDTA buffer (TE^-4^) at pH 8.0 to a final volume of 100 μL [15]. Samples were stored at -20°C and thawed at room temperature before DNA analysis.

Extracted DNA was quantified by real-time quantitative PCR using the Quantifiler human DNA quantification kit (Applied Biosystems, Foster City, CA) according to the manufacturer's protocol [16]. Sample DNA concentrations were measured in triplicate and the mean value was used. The assay was performed with the ABI 7500 Real-Time PCR System and sequence detection software v.1.2.3 (Applied Biosystems, Darmstadt, Germany).

### Statistical analysis

Analysis of variance (ANOVA) was performed to compare DNA concentrations in incinerated teeth at different temperatures, and Pearson's correlation coefficient was used to correlate spectrophotometry color values with DNA concentrations. SPSS version 23 (IBM^®^ SPSS Statistics^®^, IBM Software Group. Chicago, IL) was used for statistical analyses, considering p<0.05 as significant.

## Results

### Morphological analysis

#### Changes in the crown

**Fig 1:**
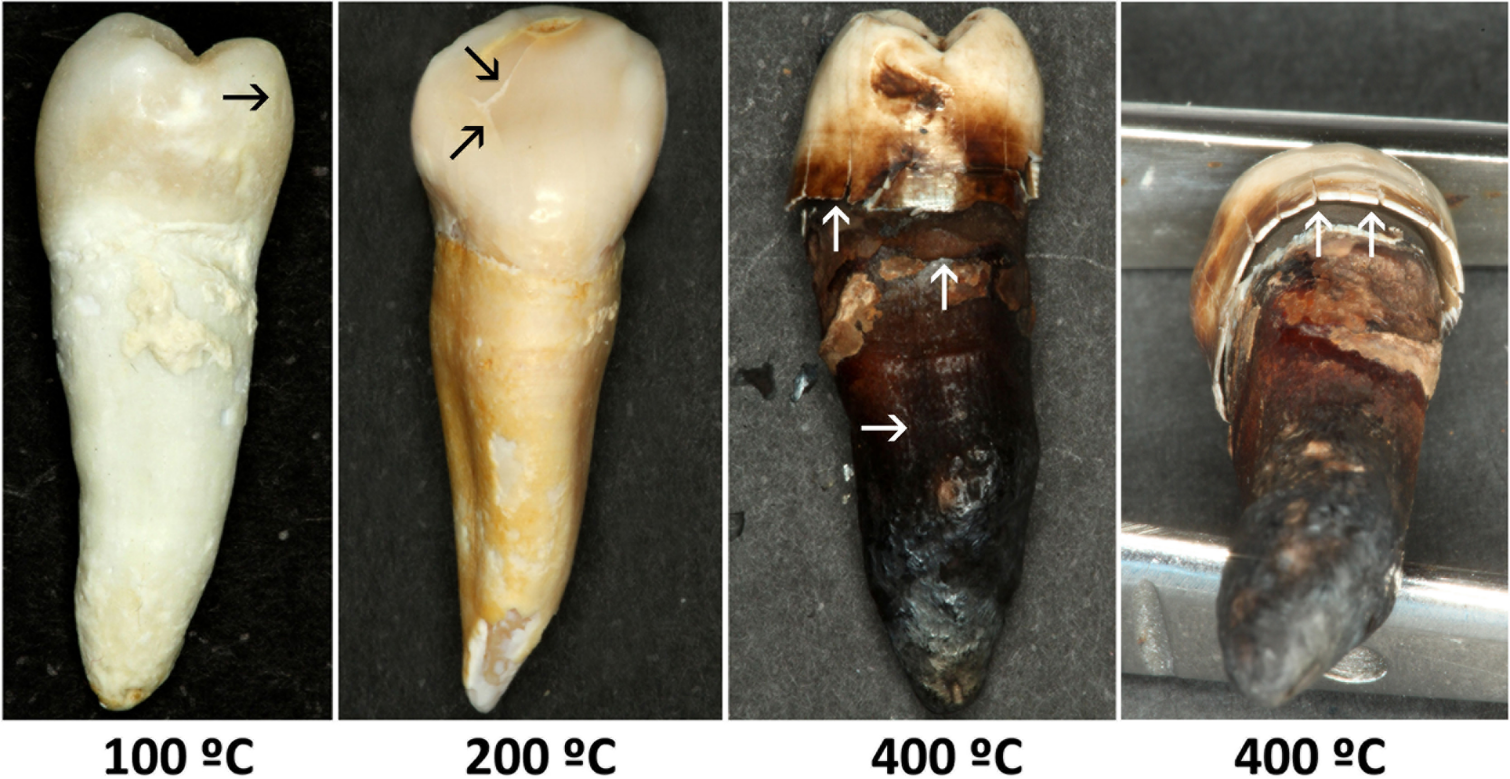
Morphologic and color changes in teeth after exposure at different temperatures (in°C). Longitudinal and transversal fissures are indicated by black arrows and longitudinal and transversal fractures by white arrows. An example of the separation between enamel and dentin is depicted in the last right photograph.

At 100°C, longitudinal fissures of the enamel were observed in 100% of the teeth and transverse fissures in 60% (Fig 1). At 200°C, longitudinal and transverse clefts of the enamel, with the onset of enamel fractures, were observed in 100% of the teeth, and a separation between enamel and dentin was detected in 40%. At 400°C, longitudinal and transverse fractures and an evident separation between enamel and dentin were observed in 100% of the teeth.

#### Root changes

At 100°C, 70% of samples presented structural changes consisting of longitudinal and transverse cracks (Fig 1). At 200°C, all teeth had longitudinal and transverse fissures, and cement fragmentation was also more frequent, occurring in 50% of the teeth. At 400°C, all teeth showed cracks and 80% fractures, while root fragmentation was observed in 20% of the teeth.

### Color measurement

In general, teeth showed a change in color from yellowish white to dark brown as the exposure temperature increased (Fig 1). At 100°C, a change in color to yellowish white was observed in 60% of samples; at 200°C, all teeth were yellowish-brown; and at 400°C, 90% were very dark brown and the remaining 10% were black.

Table 1 displays the spectrophotometry results, with data on CIELAB system parameters (L*, a*, and b* values) and WI and YI index scores at each temperature studied.

**Table 1:** Spectrophotometric color values and DNA concentrations at different temperatures.

Temperature (°C)	L*	a*	b*	Wi	Yi	DNA concentration (ng/μL)
**Control 1**	44.15	-0.06	4.67	7.9	15.8	299.56
**Control 2**	40.73	0	2.90	7.7	10.7	160.02
**Control 3**	43.23	0.02	5.95	4.5	20.4	169.71
**Control 4**	41.83	0.54	0.52	11.6	2.7	210.53
**Control 5**	42.04	-0.18	1.76	9.9	6.1	226.96
**Control 6**	41.74	0.54	7.41	2.1	26.3	210.76
**Control 7**	43.82	0.74	4.65	6.6	17	393.62
**Control 8**	43.97	0.39	1.05	12.2	4.3	317.61
**Control 9**	42.98	0.25	3.45	8	12.5	232.58
**Control 10**	40.75	0.45	2.21	8.6	8.9	256.75
**100**	45.59	-0.85	9.81	-0.1	30	2.83
**100**	39.98	0.29	10.77	-2.2	37.3	4.87
**100**	46.35	-0.88	4.04	8.8	12.2	7.23
**100**	51.59	-0.35	4.83	10.3	14.3	7.24
**100**	41.01	-0.65	-0.88	13.22	-6.4	10.62
**100**	42.93	-0.92	4.09	7	13	3.33
**100**	41.73	-40.73	0	2.9	7.7	0.19
**100**	44.02	-0.63	4.18	7.4	13.5	2.05
**100**	57.66	0.26	3.41	17.6	9.4	0.24
**100**	47.43	-0.90	10.50	-0.7	31	0
**200**	37.02	0.24	4.20	4.5	16.7	0.00399
**200**	38.28	0.17	1.12	8.8	4.6	0.0268
**200**	36.64	0.33	3.25	5.5	13.3	0.0122
**200**	34.90	0.19	2.31	5.8	9.8	0.0066
**200**	39.59	0.19	-0.35	11.5	-1.0	0
**200**	35.74	-0.01	-2.11	11.4	-9	0.0141
**200**	38.55	0.11	1.02	9.1	4.2	0.00718
**200**	41.36	0.06	4.76	5.4	17.1	0
**200**	31.85	0.015	4.23	2.9	18.3	0.0433
**200**	31.37	0.38	1.71	5.7	8.3	0.0178
**400**	31.72	-0.08	-0.09	7.1	-0.6	0
**400**	30.30	0.03	0.05	6.3	0.3	0
**400**	25.76	0.7	0.65	4.2	3.7	0
**400**	30.99	0.53	2.53	4.2	12.2	0.000807
**400**	30.65	0.54	0.69	5.8	4.1	0.000215
**400**	28.89	0.22	1.04	4.9	5.4	0
**400**	27.11	0.02	0.50	4.7	2.5	0
**400**	27.33	-0.29	0.06	5.2	-0.3	0
**400**	30.10	0.01	0.37	5.9	1.8	0
**400**	34.67	0.16	-0.73	9.2	-2.8	0

**Fig 2:**
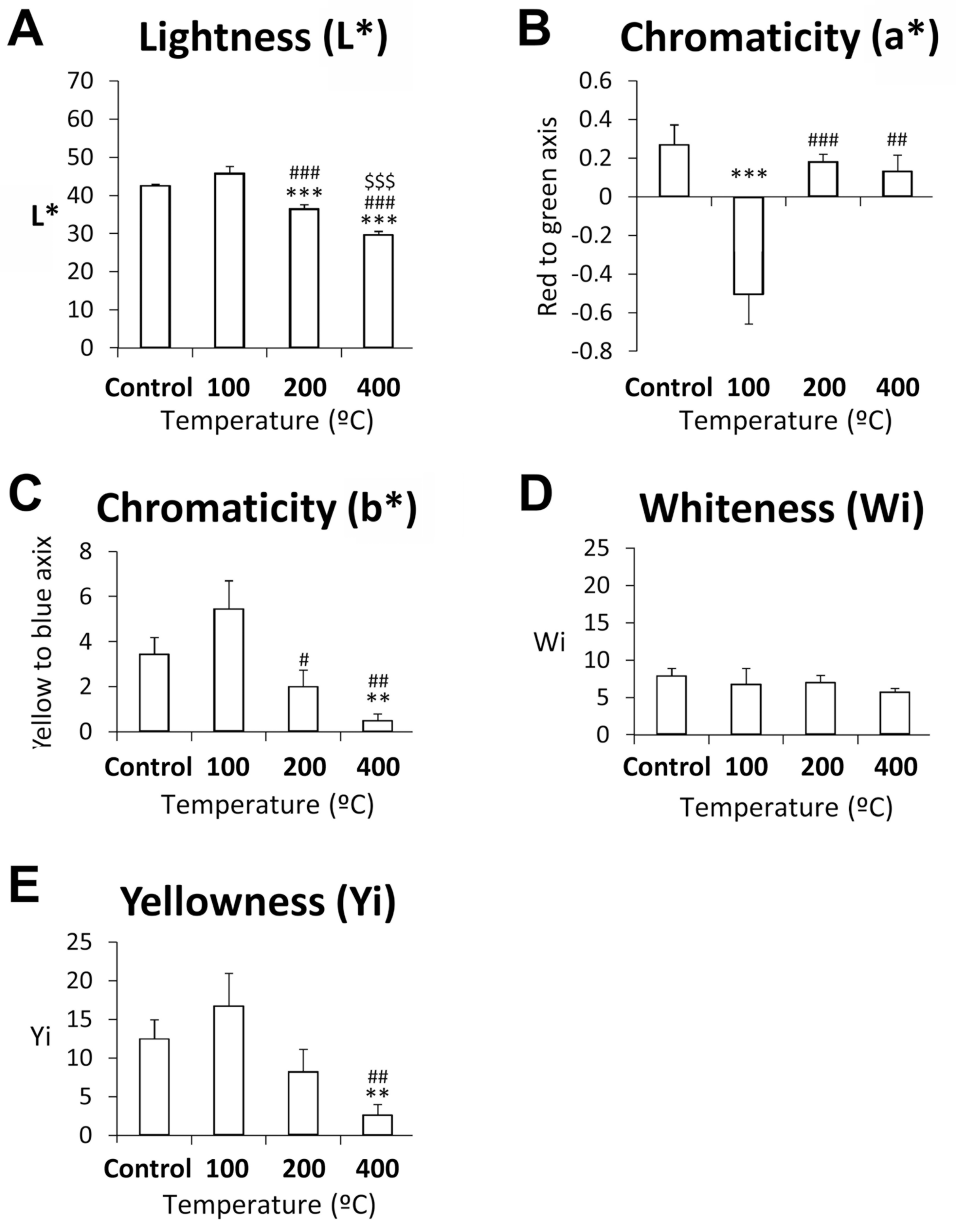
Spectrophotometric color measurements (L*, a*, b*, WI and YI) in control teeth and those heated at 100, 200, or 400°C for 60 minutes. Histograms represent means ± SEM (n = 10). ANOVA: **^/^***p<0.01/0.001 vs. control group, ^##/###^*p*<0.01/0.001 vs. 100°C, ^$ $ $^*p*<0.001 vs. 200°C.

As observed in Fig 2A, L* values diminished between 100 and 400°C, with significant differences at 200°C *versus* the control group or 100°C group and at 400° *versus* the control, 100, or 200°C groups (*p*<0.001). Chromaticity a* decreased markedly from the control group to the 100°C group, with significant differences between the 100°C group and other temperature groups (Fig 2B). A neutral red color was obtained at 200°C and 400°C and a highly saturated greenish color at 100°C. Chromaticity b* values were significantly lower at 400°C than in the control group or at 100°C, and were significantly lower at 200°C than at 100°C, with a change from a more saturated to a neutral yellow color (Fig 2C). No significant differences in WI were observed (Fig 2D). YI values were significantly lower at 400°C than in the control group or at 100°C.

### DNA quantification

As shown in Table 1, DNA could be quantified in 100% of the control group, 90% of the teeth heated at 100°C, in 80% of those heated at 200°C, and 20% of those heated at 400°C; the heating time was 60 minutes in all cases.

**Fig 3:**
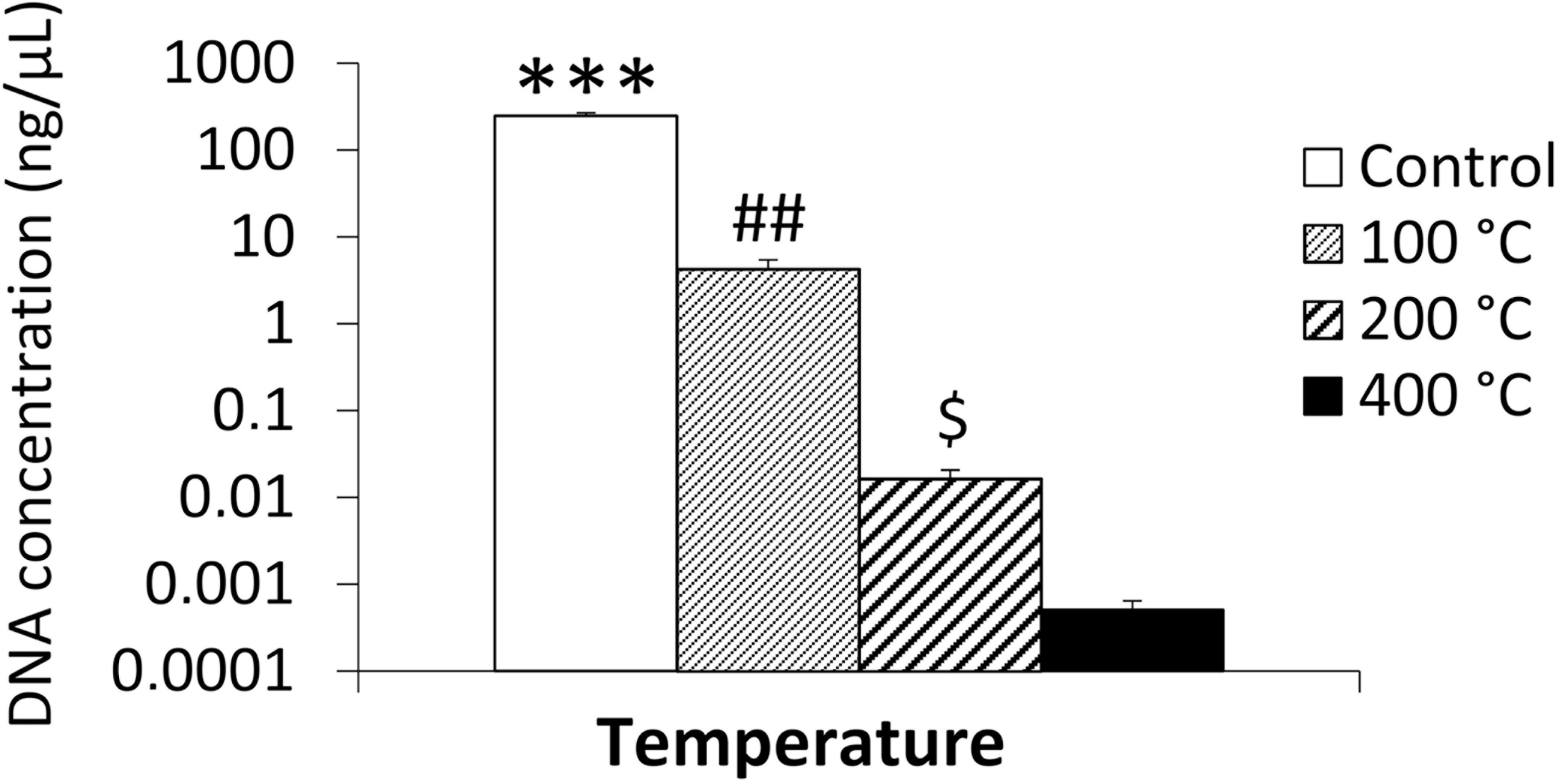
DNA concentration in control teeth and teeth heated at 100, 200, and 400°C for 60 minutes. Histograms represent means ± SEM (n = 10). ANOVA: ***p<0.001 vs.100, 200 and 400°C, ^##^*p*<0.01 vs. 200 and 400°C, ^$^*p*<0.05 vs. 400°C.

The mean DNA concentration decreased at higher temperatures, being 247.807±23.8 ng/μL in control teeth, 4.289±1.1 ng/μL in teeth heated at 100°C, 0.016±0.004 ng/μL in those heated at 200°C, and 0.00051±0.0001 ng/μL in those heated at 400°C (Fig 3). The mean DNA concentration significantly differed between the control group and all heated teeth (*p*<0.001), between teeth heated at 100°C and those heated at 200 or 400°C (*p*<0.01), and between teeth heated at 200°C (*p*<0.05) and those heated at 400°C (Fig 3). It was considered that the DNA quantification method used (Quantifiler human DNA quantification kit) was reliable to detect DNA concentration in the two samples of teeth heated at 400°C (below the 3 pg/μL stated by the manufacturer) because of the excellent characteristics of the standard curve obtained: (Slope = -3.108471; Intersection = 27.936390; R^2^ = 0.996550; Efficiency = 95–105).

### Correlation of color with DNA concentration

**Fig 4:**
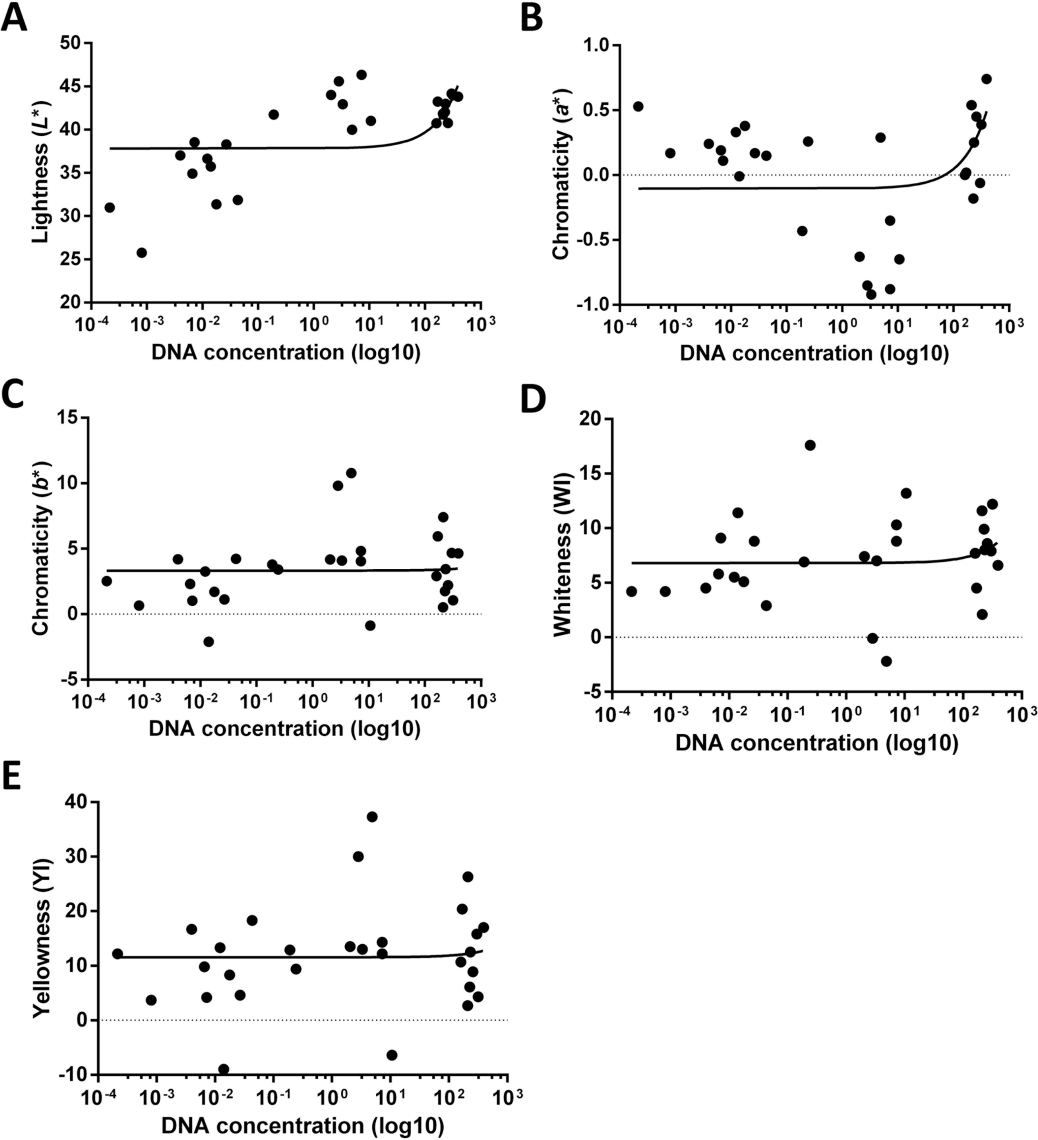
Correlation between spectrophotometric color parameters (L*, a*, b*, WI and YI) and DNA concentration in control teeth and teeth heated at 100, 200 or 400°C for 60 minutes. DNA concentration was represented on a logarithm scale (base 10).

A significant positive correlation was found between luminosity and DNA concentration (*r* = 0.4727, *p* = 0.0128), as shown in Fig 4A, and between chromaticity a* values and DNA concentration (*r* = 0.4154, *p* = 0.0250), as shown in Fig 4B. Higher luminosity and chromaticity a* values were correlated with greater DNA concentration. However, as depicted in Fig 4C, 4D and 4E, no correlation was found between DNA concentration and chromaticity b* or whiteness or yellowing indexes.

## Discussion

After an intense fire or explosion, teeth often represent the only evidence available for forensic identification [17]. In this study of the effects on teeth of heating at high temperatures for one hour, dental DNA concentrations were found to be lower at higher temperatures and to be significantly correlated with luminosity and chromaticity a* color values of the tooth surface. Most studies on these effects have exposed teeth to high temperatures for a much shorter period, but a longer duration was selected in the present study given the multiple circumstances in which this can occur, including accidents in remote locations and intentional burning, among others [18–20].

Visual analysis has been used in most investigations of changes in tooth color at high temperatures [21–23], despite the availability of more objective and accurate techniques such as spectrophotometry or spectroradiometry [24]. These techniques are widely used in restorative dentistry but not in forensic odontology, unfortunately, with age estimation being one of its few applications [25,26]. However, it was recently reported that spectrophotometric color measurements of incinerated teeth offer highly accurate estimates of the temperature of exposure [7].

The present study showed that DNA concentrations in teeth decrease with higher increases in temperature. The sample groups were homogeneous in mean age and sex, although it has been observed that the DNA concentration in teeth is not influenced by age or sex [27]. Other authors have examined the resistance of dental DNA to the action of heat [9–11, 19], and Alvarez et al [10] and Tsuchimochi et al [11] quantified DNA after 10 minutes of exposure to temperatures of 200° C and 300°C, respectively. Garriga et al [12] obtained similar results with exposure times of 1 to 15 minutes and temperatures of 100° to 700° C, being able to extract DNA from almost all samples. In the present study, only small concentrations of DNA were obtained in teeth heated at 200° C or 400° C, likely attributable to the longer exposure of 60 minutes.

To our best knowledge, this is the first time that the color of teeth burned at different temperatures has been related to the extraction and quantification of the DNA they contain. We found that DNA concentrations were significantly and positively correlated with the luminosity and with chromaticity a* values. It may therefore prove possible to predict the possibility of extracting DNA from burned dental remains based on colorimetric variables, and the color of teeth may assist in the selection of promising specimens for DNA extraction.

One study limitation is that it was performed in teeth without the protection provided by the alveolar bone, maxillary bone, or soft tissues. Ellinghan et al [28] observed that soft tissue affects bone changes by slowing combustion at the beginning and accelerating it at the end. Rees et al [21] extracted quantifiable DNA at temperatures up to 525°C from *SusScrofa* molars protected by the alveolar bone and up to 625°C from those protected by the whole head, highlighting the protection offered by muscle and other tissues against sample degradation. Hence, besides temperature, consideration should also be taken of differences between protected and unprotected body areas. A further study limitation is that the effects of incineration were investigated in a controlled laboratory environment, and no account was taken of the influence of accelerant type or fire extinction method, among other relevant factors, including the duration of exposure.

In conclusion, dental color, specifically luminosity and chromaticity a* values, were significantly correlated with DNA concentrations in burned teeth. It may prove possible to predict the possibility of DNA extraction from burned teeth based on the spectrophometric study of their color. Further comparisons are warranted to verify the surprisingly large difference in DNA concentration between teeth stored at room temperature and those heated at 100°C. Studies are also needed to establish the effects of other exposure times.
